# Cancer Screening Literacy among Vietnamese Population: Does Annual Checkup Improve Cancer Screening Literacy?

**DOI:** 10.31557/APJCP.2021.22.3.927

**Published:** 2021-03

**Authors:** Sangchul Yoon, Kun Wang, Yan Luo, Jongwook Lee, Jessica Neese, Hee Yun Lee

**Affiliations:** 1 *Yonsei University, Republic of Korea. *; 2 *School of Social Work, The University of Alabama, Tuscaloosa, AL, United States. *; 3 *Department of Global Health and Population, Harvard T.H. Chan School of Public Health, Republic of Korea. *

**Keywords:** Cancer literacy, colorectal cancer, breast cancer, cervical cancer, HPV-related cancers, Vietnamese, cancer

## Abstract

**Background:**

Colorectal, breast, and cervical cancers disproportionately impact the Vietnamese population. However, research on cancer prevention among this population was very limited. The purpose of this study is to examine the cancer screening literacy levels for these three types of cancers among rural Vietnamese and investigate correlates of cancer screening literacy.

**Methods:**

A sample of 226 Vietnamese men and women aged 25-70 years old was recruited from rural Vietnam and finished a self-administered questionnaire. Andersen’s Behavioral Model was used to guide this cross-sectional study to identify modifiable variables. Bivariate analysis was used to explore the relationship between demographic factors and cancer screening literacy levels. Multiple linear regressions were also used to identify significant factors for cancer literacy levels.

**Results:**

Cancer screening literacy levels of Vietnamese men and women were low regarding all three types of cancers, especially HPV symptom questions. Only about 24% of women answered correctly on “most people with genital HPV have no visible signs/symptoms” and less than 18% answered correctly on “I can transmit HPV to my partner(s) even if I have no HPV symptoms.” Findings suggested that having an annual checkup was associated with higher colorectal (β=.15, p<.05), breast (β=.25, p<.001), and cervical (β=.18, p<.01) cancer screening literacy.

**Conclusions:**

Public health efforts should focus on encouraging annual checkups in the Vietnamese population. During the annual checkup, health care professionals should educate patients about importance of cancer screening and provide recommendations for regular cancer screenings to reduce cancer health disparities.

## Introduction

Colorectal cancer, breast cancer, and cervical cancer are three of the most prevalent cancers in Vietnam (The Global Cancer Observatory, 2018). In 2018, 7,856 Vietnamese died from colorectal cancer, 6,103 Vietnamese women died from breast cancer, and 2,420 women died from cervical cancer (Bruni et al., 2018; The Global Cancer Observatory, 2018). Due to the effectiveness on the early detections (Zauber, 2015; American Cancer Society, 2019), cancer screenings have been commonly recommended for early detections and treatments. Common cancer screening modalities include sigmoidoscopy, colonoscopy, and fecal occult blood test for colorectal cancer, mammogram for breast cancer, and Pap test and human papilloma virus (HPV) vaccination for cervical cancer. However, little is known about cancer screening among Vietnamese. 

A country profile of Vietnam from the World Health Organization (2014) reported that cancer screenings and early detections of colorectal cancer, breast cancer, and cervical cancer were not generally available at the public primary health care level, despite that these cancer screenings are available and effective in detecting precancers. Even in places where cancer screenings and early detections were available, previous studies indicated that low cancer literacy or cancer knowledge was a significant barrier to obtaining cancer screenings (Carpenter and Colwell, 1995; Kandula et al., 2006; Lee and Vang, 2010). Kandula (2006) reported that low rates of colorectal, cervical, and breast cancer screening were associated with the belief that cancer screening was not needed unless the presence of symptoms. Comparatively, Talley (2017) suggested that patients with higher cancer literacy were more likely to be motivated to get cancer screenings. Besides, a variety of studies on specific types of cancers, such as breast cancer (Pearlman et al., 1999) and cervical cancer (Pearlman et al., 1999; Lee, 2000; Juon et al., 2003), also supported the strong relationship between cancer literacy and the uptakes of cancer screenings. A more recent study by Lee (2016) indicated that cancer literacy was a mediator for cancer screening behaviors among Korean adults. Specifically, the effects of use of eastern medicine and self-rated health status were fully mediated by cancer literacy. Therefore, in order to narrow the cancer screening gap, interventions aiming to improve cancer literacy are needed (Lee et al., 2015). 

Although cancer literacy plays a significant role in promoting cancer screening rates, only two studies have explored factors associated with cancer literacy for Vietnamese population. Zambrana (2015) indicated the strong relationship between cancer literacy and family communication, higher educational attainment, employment, and family self-reported health status, while Lindau (2002) suggested that poor health literacy was a risk factor of low cancer screening knowledge. To our knowledge, there is limited research examining the related factors for Vietnamese cancer screening literacy. The present study aims to: (1) examine the levels of colorectal cancer literacy, breast cancer literacy, and cervical cancer literacy among Vietnamese and (2) investigate associated factors with colorectal cancer literacy, breast cancer literacy, and cervical cancer literacy, especially explore the relationship between annual health checkups and these three types of literacy. This study would contribute to the very limited literature of cancer screening literacy study among Vietnamese, and provide more insights for future research, practice and policy for this population.

## Materials and Methods

*Research Design and Data Collection*

This study was part of a larger research project on health and cancer screening literacy in Vietnam. The research protocol was approved by the Institutional Review Board (IRB) at the authors’ university. A convenient purposive sample of 226 Vietnamese males and females aged 25 to 70 years old were recruited from rural Vietnam in Quang Tri Province. A 10-page self-administered questionnaire was used to collect data. It took 25-30 minutes to finish the survey, and all participants were given $5 equivalencies to appreciate their time commitment. 

*Instruments*

*Dependent variables*

The outcome variables in this study were colorectal cancer literacy, breast cancer literacy, and cervical cancer literacy. Colorectal cancer literacy data was collected among both the male and female participants using a 7-item scale developed from the American Cancer Society’s Colorectal Cancer Screening Guideline (American Cancer Society, 2016b) and Colorectal Cancer Risk Factors (American Cancer Society, 2016a). Breast cancer literacy was measured by a 5-item scale based on the American Cancer Society’s Guidelines for Breast Cancer Screening (2003), and only female participants were investigated in this section. Cervical cancer literacy was measured by a 5-item scale on cervical cancer screening and risk factors, and a 7-item scale on HPV and HPV knowledge developed from the Center for Disease Control and Prevention (2013). Cervical cancer literacy was only applicable to female participants. All the items were dichotomized (0=False, 1=True), and the sum of the scores was used to measure participants’ cancer literacy.

*Independent variables*

Andersen’s Behavioral Model was the chosen framework to guide the study. Based on this model, independent variables were categorized into predisposing variables, enabling variables, and need variables. Gender was only analyzed in the colorectal cancer literacy model while all other variables were applied to the three cancer literacy models. 

Predisposing variables. Age, gender, marital status, and education level were selected as predisposing variables in this study. Age was a continuous variable. Gender (0=male, 1=female), marital status (0=not married or partnered, 1=married or partnered), and education level (0=less than a bachelor’s degree, 1=bachelor’s degree or higher) were measured dichotomously. 

Enabling variables. Employment status, having health insurance, and annual checkup were categorized as enabling variables. All the three enabling variables were dichotomized—employment status (0=unemployed, 1=employed), having health insurance (0=no, 1=yes), and having an annual checkup (0=no, 1=yes).

Need variables. Family cancer history and self-rated health scores were need variables. Binary-choice question (0=no, 1=yes) was employed to measure whether participants had family cancer history. Self-rated health score was analyzed as a continuous variable and measured by a 5-point Likert scale and recoded to 0-4 (0=poor, 1=fair, 2=good, 3=very good, 4=excellent). 

*Data Analysis*

Univariate analysis was utilized to examine the sociodemographic characteristics of the sample and analyze cancer literacy descriptively. Bivariate analysis (chi-square test and t-test) was employed to test the relationship between sociodemographic characteristics and different kinds of cancer literacy. Finally, multiple linear regressions were conducted on all independent variables to identify associated factors for colorectal cancer literacy, breast cancer literacy, and cervical cancer literacy, respectively. All the analyses in this study were completed in SPSS version 25.0.

## Results

*Sociodemographic Characteristics of the Sample*

As we can see from Table 1, among all the 226 participants aged from 29 to 67 years old, 85.4% were female, and 14.6% were male. The mean age of the total participants was 41.6 years old, whereas the mean age of the female participants was 40.84 years old. Among the total participants, the majority were married or partnered and employed, and had health insurance, and did not have a bachelor’s degree or higher and family cancer history. More than half of participants had an annual checkup and a mean self-rated health score of 2.05. Similar characteristics were found among female participants where the majority were also married or partnered and employed, and had health insurance, and did not have a bachelor’s degree or higher and family cancer history. Above half of female participants had an annual checkup and a mean health score of 2.06.

As Table 1 indicates, significant differences of participants’ colorectal cancer literacy were found on education level and having annual checkup. Concerning breast cancer literacy, notable differences existed on employment status, educational level and having annual checkup. Finally, cervical cancer literacy was also significantly different according to employment status, education level and having annual checkup.

*Descriptive Analysis of Cancer Literacy*

Table 2 showed that participants had a relatively low literacy of colorectal cancer, breast cancer, and cervical cancer. As to the colorectal cancer literacy, correction rates to “To screen colorectal cancer or find polyps, one should take flexible Sigmoidoscopy every 5 years” and “To screen colorectal cancer or find polyps, one should take flexible Colonoscopy every 10 years” were only about 32.2% and 12.3%, respectively. With respects to breast cancer literacy, less than half (42.5%) could give the correct answer to “Women whose close blood relatives have breast cancer have a higher risk for this disease.” A total of 12 items questioned participants about cervical cancer literacy. However, less than one third of participants could give the correct answer to “Birth control pills can increase the risk of getting cervical cancer” (29.0%), “Most people with genital HPV have no visible signs or symptoms” (23.8%) and “I can transmit HPV to my partner(s) even if I have no HPV symptoms” (17.6%).

*Multiple Linear Regression Analysis on Cancer Literacy*

Three multiple linear regressions were conducted to assess factors predicting colorectal cancer literacy, breast cancer literacy, and cervical cancer literacy, respectively (Table 3). Education level (β=0.219, SE=0.373, p<0.01) and having annual checkup (β=0.146, SE=.0289, p<0.05) were significant indicators of colorectal cancer literacy. In the breast cancer literacy model, education level (β=0.183, SE=0.350, p<0.05), having annual checkup (β=0.245, SE=0.272, p<0.001), and self-rated health score (β=0.160, SE=0.224, p<0.05) were significant variables. In the cervical cancer literacy model, age (β=-.149, SE=0.042, p<0.05), education level (β=0.283, SE=0.637, p<0.001), and having annual checkup (β=0.182, SE=.496, p<0.01) were found to be significant predictors among female participants.

**Table 1:** Sociodemographic Characteristics of the Samples

	Total			Women only				
	N (%)	Colorectal cancer		N (%)	Breast cancer		Cervical cancer	
		Mean (SD)	p-value		Mean (SD)	p-value	Mean (SD)	p-value
Gender			0.541					
Male	33 (14.6)	3.30 (2.24)						
Female	193 (85.4)	3.06 (2.06)						
Marital status			0.159			0.732		0.412
Others	17 (7.5)	2.41 (2.18)		16 (8.3)	2.44 (2.03)		6.25 (4.11)	
Married/ Partnered	209 (92.5)	3.15 (2.07)		177 (91.7)	2.60 (1.85)		5.51 (3.36)	
Employment status			0.104			0.016		0.008
Yes	204 (90.3)	2.41 (2.13)		172 (89.1)	2.70 (1.85)		5.80 (3.35)	
No	22 (9.7)	3.17 (2.07)		21 (10.9)	1.67 (1.74)		3.71 (3.55)	
Education level			0			0		0
< Bachelor’s degree	184 (81.4)	2.84 (2.07)		159 (82.4)	2.37 (1.83)		5.03 (3.39)	
>= Bachelor’s degree	42 (18.6)	4.24 (1.78)		34 (17.6)	3.62 (1.71)		8.12 (2.29)	
Family cancer history			0.615			0.727		0.34
Yes	54 (23.9)	3.22 (1.88)		49 (25.4)	2.51 (1.88)		5.98 (3.57)	
No	172 (76.1)	3.06 (2.15)		144 (74.6)	2.62 (1.86)		5.44 (3.38)	
Having health insurance			0.286			0.116		0.139
Yes	182 (80.5)	3.17 (2.04)		158 (81.9)	2.69 (1.88)		5.75 (3.42)	
No	44 (19.5)	2.80 (2.26)		35 (18.1)	2.14 (1.73)		4.80 (3.41)	
Having annual checkup			0.007			0		0.004
Yes	122 (54.0)	3.44 (1.99)		108 (56.0)	3.06 (1.73)		6.20 (3.28)	
No	104 (46.0)	2.69 (2.14)		85 (44.0)	2.00 (1.86)		4.78 (3.46)	
	Mean (SD)			Mean (SD)				
Age (29-67)	41.60 (5.88)			40.84 (5.67)				
Self-rated health (0-4)	2.05 (.58)			2.06 (.60)				

**Table 2:** Descriptive Analysis on Cancer Literacy

Knowledge about Colorectal Cancer (N=226, M=3.10, SD=2.09)	Participants answered correctly	%
1. Both men and women at age of 50 should begin colorectal cancer screening.	103	45.6
2. To screen colorectal cancer or find polyps, one should take flexible Sigmoidoscopy every 5 years.	73	32.3
3. To screen colorectal cancer or find polyps, one should take flexible Colonoscopy every 10 years.	28	12.4
4. To detect colorectal cancer, one should take fecal occult blood test (FOBT) every year.	130	57.5
5. Obesity raises the risk of colon cancer in both men and women.	89	39.4
6. Long-term smokers are more likely than non-smokers to develop and die from colorectal cancer.	131	58
7. Colorectal cancer has been linked to the heavy use of alcohol.	146	64.6
Knowledge about Breast Cancer (N=193, M=2.59, SD=1.86)	Participants answered correctly	%
1. Yearly mammograms are recommended starting at age 40 and continuing for as long as a woman is in good health.	102	52.8
2. Clinical breast exam (CBE) is recommended about every 3 years for women in their 20s and 30s and every year for women 40 and over.	102	52.8
3. Breast self-exam (BSE) is an option for women starting in their 20s.	103	53.4
4. The risk of developing breast cancer increases as getting older.	111	57.5
5. Women whose close blood relatives have breast cancer have a higher risk for this disease.	82	42.5
Knowledge About Cervical Cancer Screening and HPV (N=193, M=5.58, SD=3.43)	Participants answered correctly	%
1. All women should begin cervical cancer screening such as Pap test at age 21.	121	62.7
2. Beginning at age 30, women who have had 3 normal Pap test results in a row may get screened every 2 to 3 years.	87	45.1
3. Birth control pills can increase the risk of getting cervical cancer.	56	29
4. I think Pap testing is necessary for asymptomatic women.	116	60.1
5. I believe Pap test can help detect cervical cancer earlier.	148	76.7
6. Human papilloma virus (HPV) infection can cause cervical cancer.	96	49.7
7. Most people with genital HPV have no visible signs or symptoms.	46	23.8
8. If a woman’s Pap test is normal, she does not have HPV.	82	42.5
9. Pap tests will almost always detect HPV.	99	51.3
10. A negative test for HPV means that you do not have HPV.	100	51.8
11. A vaccine exists to prevent HPV infection.	91	47.2
12. I can transmit HPV to my partner(s) even if I have no HPV symptoms.	34	17.6

**Table 3:** Multiple Linear Regression of Cancer Literacy

Variables	Colorectal Cancer Literacy	Breast Cancer Literacy	Cervical Cancer Literacy
	β (SE)	β (SE)	β (SE)
Gender (Ref=male)	-0.056 (0.412)	-	-
Age (29-67)	-0.073 (0.025)	-0.019 (0.023)	-0.149* (0.042)
Marital status (Ref=not married or partnered)	0.112 (0.51)	0.058 (0.456)	-0.014 (0.83)
Employment status (Ref=not employed)	0.04 (0.471)	0.09 (0.418)	0.098 (0.762)
Education level (Ref<bachelor’s degree)	0.219** (0.373)	0.183* (0.350)	0.283*** (0.637)
Having health insurance (Ref=no)	-0.015 (0.364)	-0.007 (0.349)	-0.007 (0.637)
Annual checkup (Ref=no)	0.146* (0.146)	0.245*** (0.272)	0.182** (0.496)
Family cancer history (Ref=no)	0.024 (0.323)	-0.025 (0.297)	0.033 (0.542)
Self-rated health score (0-4)	0.05 (0.249)	0.160* (0.224)	0.062 (0.408)
Number of observations	226	193	193
R^2^	0.111	0.171	0.186
F	2.998**	4.749***	5.244***

## Discussion

Guided by Andersen’s Behavioral Model, the current study examined the literacy levels of three leading cancers – colorectal cancer, breast cancer and cervical cancer, investigated associated factors, and explored the relationship between annual health checkups and these three types of cancer literacy. 

Results in this study indicated low levels of cervical cancer and HPV literacy regarding symptoms of HPV. A shockingly low number of participants (23.8%) were aware that genital HPV could have no signs or symptoms, and even less (17.6%) knew HPV could be transmitted even without symptoms present, which literature suggests is common among this population. The current study findings were lower than previous studies have indicated; Yi and colleagues reported that 45% of their study’s sample of Vietnamese mothers residing in the U.S. were aware that HPV could be asymptomatic (Yi et al., 2013). Lack of knowledge about the absence of symptoms can result in a false sense of security regarding contracting an HPV infection. Prevention of HPV is possible with the uptake of the HPV vaccine; however, literature suggests Asian Americans have low knowledge of the vaccine. According to Lee (2015), nearly a quarter of their study’s participants of Asian American undergraduates had prior knowledge of the vaccine; yet just over half of those individuals were aware the vaccine prevented HPV infection and cervical cancer. 

Findings indicated that both male and female participants have relatively low colorectal cancer literacy, with only 45.6% knowing which age to begin preventative screening measures, and even less knowing how often to receive a sigmoidoscopy, 39.4% reported every five years and 32.2% reported every ten years. This low literacy is not uncommon in the Vietnamese population; according to Le (2014), only 40.2% of Vietnamese immigrant participants had heard about colon polyps, which are growths on the colon that a sigmoidoscopy detects. Le (2014) also reported participants were not aware of colorectal screening opportunities and a study completed by Kim (2015), concluded that only 30% of Vietnamese immigrant participants initiated uptake and completed a sigmoidoscopy in the last five years. 

In comparison to previous literature, the current study’s findings are significantly higher regarding breast cancer literacy. When asked at what age a woman is recommended to start screening measures and how often screening should be completed, above half (52.8%) of participants in the current study answered correctly compared to less than 40% of participants in Yi (2005) study. Moreover, 42.5% of participants in the present study compared to only 26% of Vietnamese and Cambodian women in a previous study (Phipps et al., 1999) were aware that having a family history of cancer increased their own risk of cancer. According to Petrisek (2000), participants who had a known family history of cancer were more aware of their cancer risk, and it resulted in more proactive measures and frequent uptake of cancer screenings. 

Factors such as education and annual checkup were found to be significant in association with colorectal cancer literacy. Although the study of Le et al., (2014) did not include an annual checkup as a variable specifically, findings indicated that having a regular healthcare provider was a necessary means to obtaining colorectal cancer knowledge. Of the Vietnamese participants who took part in the study, 45% had a regular healthcare provider, but only 39.8% had a prior discussion with their healthcare provider about colorectal cancer; however, 86% stated they would obtain a screening at their provider’s recommendation. These findings suggest a gap in communication with healthcare providers and further exploration in determining communication barriers exists. 

Employment, education, and annual checkup were identified to be significant factors in determining cervical cancer and breast cancer literacy in the present study. Age was determined to be an indicator of cervical cancer literacy only. Lee (2015) determined age and use of gynecological services along with HPV literacy had associations with HPV vaccine literacy. Using gynecological services in this study can correlate to having an annual checkup since both theoretically include physician recommendations for preventive services, like the HPV vaccine, and physician examinations, like the Pap test, which both have the ability to prevent HPV-related cancers. Breast cancer screening has similar factors, including higher education, age, regular healthcare facility, and regular physician, having a positive association, and being unemployed was considered to have a negative association (Lee-Lin and Menon, 2005). 

To our knowledge, the current study is one of the first to provide empirical evidence on the importance of annual health checkup regarding colorectal, breast, and cervical cancer literacy. In place of annual health checkup, previous literature explored other enabling factors such as health insurance, regular healthcare facility, and regular healthcare provider and its significance in determining colorectal cancer, breast cancer, and HPV literacy and cancer screening (Lee-Lin and Menon, 2005; Yi et al., 2013; Kim et al., 2014; Le et al., 2014; Kim et al., 2015; Lee et al., 2015). Similar to an annual health checkup, the variable of physician’s recommendation also has the potential to increase an individual’s literacy on cancer screening. However, a barrier to both is the issue of individuals only seeking their healthcare provider when experiencing a healthcare problem and not using services in a proactive manner (Le et al., 2014). 

*Limitations*

Although the present study provides valuable insight into colorectal cancer, breast cancer, and cervical cancer literacy of individuals in Vietnam, there are some limitations. Firstly, due to the gap of cancer literacy studies taken place in Vietnam, literature used to enforce findings of the present study were majority conducted in the United States either using Vietnamese Immigrants or Vietnamese Americans; however, the present study will add to existing Vietnamese literature and create a foundation of cancer literacy literature. Secondly, in this cross-sectional study, only correlation could be identified. It is unclear whether having annual checkups increase cancer literacy or high cancer literacy leads to the annual checkup. More future longitudinal studies are needed to further explore the causal relationship. Finally, participants in this study were recruited from the rural areas in Quang Tri Province, Vietnam. Thus, the generalization of this study results in the general Vietnamese population may not be appropriate. 

*Implications for Practice and Research*

Despite the limitations, the current study provides significant implications for healthcare practice and research. The findings highlighted the importance of annual health checkups on colorectal cancer, breast cancer, and cervical cancer literacy for the Vietnamese population and suggest educating individuals on the importance of routine checkups and how they increase knowledge of various preventative measures and promote self-efficacy. Previous literature has identified barriers associated with cancer screening, that the present study did not consider that future exploration is needed. These barriers include, lack of perceived health problems, challenge with medical terminology, and physician recommendation, which can all have an impact on health help-seeking behaviors and self-efficacy in cancer screening preventative measures (Kimura et al., 2014).

In conclusion, cancer screening literacy levels of Vietnamese men and women were low regarding all three types of cancers. In particular, knowledge about HPV and HPV vaccine related to cervical cancer screening literacy were extremely low among female participants. Findings suggested that having an annual checkup was a significant indicator for cancer screening literacy of all the three types of cancers. This study is one of the first to provide empirical evidence on the importance of annual health checkup regarding colorectal, breast, and cervical cancer literacy. Public health efforts should focus on encouraging annual checkups and preventative cancer screenings in the Vietnamese population. During the annual checkup, health care professionals should educate patients about the importance of cancer screening and provide recommendations for regular cancer screenings to reduce cancer health disparities.

## Author Contribution Statement

None.
